# Potential clinical impact of T-cell lymphocyte kinetics monitoring in patients with B cell precursors acute lymphoblastic leukemia treated with blinatumomab: a single-center experience

**DOI:** 10.3389/fimmu.2023.1195734

**Published:** 2023-09-22

**Authors:** Andrea Duminuco, Uros Markovic, Nunziatina Laura Parrinello, Luca Lo Nigro, Elisa Mauro, Calogero Vetro, Marina Parisi, Cinzia Maugeri, Paolo Fabio Fiumara, Giuseppe Milone, Alessandra Romano, Francesco Di Raimondo, Salvatore Leotta

**Affiliations:** ^1^ Division of Hematology and Bone Marrow Transplant Unit, Azienda Ospedaliero Universitaria Policlinico “G.Rodolico-San Marco”, Catania, Italy; ^2^ Postgraduate School of Hematology, University of Catania, Catania, Italy; ^3^ Division of Hematology with Bone Marrow Transplant, Istituto Oncologico del Mediterraneo, Viagrande, Italy; ^4^ Center of Pediatric Hematology Oncology, Azienda Ospedaliero Universitaria Policlinico “G.Rodolico-San Marco”, Catania, Italy; ^5^ Dipartimento di Specialità Medico-Chirurgiche, CHIRMED, Sezione di Ematologia, University of Catania, Catania, Italy

**Keywords:** blinatumomab, T-cell kinetics, minimal residual disease, acute lymphoblastic leukemia, cytokines, MDSCs

## Abstract

Blinatumomab is a bispecific anti-CD3 and anti-CD19 antibody that acts as a T-cell engager: by binding CD19+ lymphoblasts, blinatumomab recruits cytotoxic CD3+ T-lymphocytes to target the cancer cells. Here we describe seven different patients affected by B-cell precursor acute lymphoblastic leukemia (Bcp-ALL) and treated with blinatumomab, on which we evaluated the potential association between the amount of different T-cells subsets and deep molecular response after the first cycle, identified as a complete remission in the absence of minimal residual disease (CR/MRD). The immune-system effector cells studied were CD3+, CD4+ effector memory (T4-EM), CD8+ effector memory (T8-EM), and T-regulatory (T-reg) lymphocytes, and myeloid-derived suppressor cells (MDSC). Measurements were performed in the peripheral blood using flow cytometry of the peripheral blood at baseline and after the first cycle of blinatumomab. The first results show that patients with a higher proportion of baseline T-lymphocytes achieved MRD negativity more frequently with no statistically significant difference (p=0.06) and without differences in the subpopulation count following the first treatment. These extremely preliminary data could potentially pave the way for future studies, including larger and less heterogeneous cohorts, in order to assess the T-cell kinetics in a specific set of patients with potential synergy effects in targeting myeloid-derived suppressor cells (MDSC), commonly known to have an immune evasion mechanism in Bcp-ALL.

## 1 Introduction

Treating B-cell precursor acute lymphoblastic-B cell leukemia (Bcp-ALL) is historically a challenge, above all in adult-age patients, where the hematopoietic stem cell transplant (HSCT) represented a curative choice. The use of chemotherapic regimens based on pediatric-inspired schemes has changed the landscape, improving the outcomes of these patients (1, 2). Some studies over the years suggested for some categories of patients no survival advantage from HSCT in first complete remission compared to the intensive or pediatric-based chemotherapy regimens alone or combined with tyrosine-kinase inhibitors (TKIs) for Philadelphia-chromosome positive (Ph’+) Bcp-ALL (3, 4). On the other side, the introduction of immunotherapy with monoclonal antibodies opened up a new treatment chapter for Bcp-ALL patients, above all when used as a consolidation treatment for those with measurable residual disease (MRD) after chemotherapy treatment and in case of relapse. In this scenario, blinatumomab represents the first bispecific anti-CD3 and anti-CD19 monoclonal antibody that acts as a T-cell engager recruiting cytotoxic CD3+ T-lymphocytes and directing them to attack CD19+ lymphoblastic cells. In a phase 3 study in patients with relapsed/refractory (R/R) Bcp-ALL, blinatumomab, compared to standard “rescue” chemotherapy, demonstrated superiority both in terms of complete response rate (44% vs. 25%) and overall survival (7.7 months vs. 4 months) (5). Although it is evident that the mechanism of action of blinatumomab involves the patient’s immune system, confirmation of the potential role of lymphocyte cytokine kinetics is still lacking. Moreover, it is not well understood which immune mediators (immune-effector cells and cytokines) play a major role in determining the response to blinatumomab. MDSCs cover a central role and are significantly elevated in peripheral blood and bone marrow of Bcp-ALL patients, correlated with the clinical therapeutic responses through an initially well-described mechanism of immune evasion of tumor cells (6).

Starting from these premises, in this case series, we describe the treatment response to blinatumomab in 7 Bcp-ALL patients with unfavorable characteristics and collect data regarding specific immunological markers associated with peripheral blood T-cell lymphocytes as potential predictive factors of deep molecular response to blinatumomab. All patients have provided written informed consent and were evaluated with peripheral blood flow cytometry according to our center’s internal guidelines due to the specific targeting mechanism of the bispecific antibody.

## 2 Case-series presentation

A schematic representation of the cases is reported in **Table 1**.

**Table 1:** A brief summary of the 7 patients described in the text.

Case N.	Patients and ALL’s features at diagnosis	Previous treatments for ALL	Status of disease at blinatumomab treatment	Concurrent treatment and n. of cycles	Type of response	Outcome
1	55-year-old female with Ph’+ ALL-B	Dasatinib and CS; ponatinib; chemotherapy based on the hyper-CVAD scheme	Active disease	2 cycles	CR with MRD (1^st^ cycle) CR and MRD- (2^nd^ cycle)	HSCT consolidation after blinatumomab, close follow-up started, without signs of disease after five months
2	57-year-old male with Ph’- ALL-B	Polychemotherapy scheme for 5 cycles	CR with MRD	3 cycles	CR and MRD- (1^st^ cycle)	HSCT consolidation after blinatumomab, complicated by GVHD and maintaining CR after six months
3	18-year-old female with Ph’- ALL-B	Polychemotherapy scheme with CR for 5 years. Vincristine, idarubicin, and chrysantaspase scheme for 1^st^ relapse	CR with MRD	2 cycles	CR and MRD- (1^st^ cycle)	HSCT consolidation after blinatumomab, without signs of relapse after 3 years
4	42-year-old female with Ph’+ ALL-B	Dasatinib and CS; polychemotherapy scheme for MRD, followed by consolidation with HSCT. Ponatinib and DLI for early relapse; 3 cycles of polychemotherapy scheme	Active disease	2 cycles, associated with DLI	CR with MRD (1^st^ cycle) Relapse after 2^nd^ cycle	Treated with inotuzumab ozogamicin, venetoclax, asciminib, until death for disease’s progression
5	36-year-old female with Ph’+ ALL-B	Dasatinib and CS; methotrexate and high-dose cytarabine consolidated by HSCT and ponatinib as maintenance therapy. For subsequent relapse, polychemotherapy	CR with MRD	5 cycles, associated with DLI	CR with MRD (1^st^ cycle) CR and MRD- (2^nd^ cycle) Relapse after 5^th^ cycle	Treated with inotuzumab ozogamicin associated with ponatinib, ASP-based polychemotherapy, venetoclax, until death for disease’s progression
6	14-years-old male with Ph’- ALL-B	Polychemotherapy protocol for induction of remission; 6-mercaptopurine and MTX as maintenance	CR with MRD	3 cycles	CR and MRD- (1^st^ cycle)	Close follow-up, without signs of relapse after 2 years
7	16-years-old male with Ph’- ALL-B	Polychemotherapy scheme with CR for 8 months. Vincristine, mitoxantrone, and ASP for relapse	CR with MRD	2 cycles	CR and MRD- (1^st^ cycle)	Close follow-up, without signs of relapse after 1 year

ALL-B, acute lymphoblastic B-leukemia; CS, corticosteroids; CR, complete remission; MRD, minimal residual disease; HSCT, hematopoietic stem cell transplant; ASP, asparaginase; DLI, donor lymphocyte infusion.

### 2.1 Case n. 1

A 53-year-old female patient diagnosed with Ph+ Bcp-ALL was being treated in another center for the first two years from diagnosis. She received a first-line therapy based on a second-generation tyrosine kinase inhibitor (dasatinib 100 mg daily) and steroids between March 2020 and October 2020, achieving complete hematological remission (CR), i.e., blast cells in the bone marrow (BM) <5% without evidence of extramedullary disease (EMD). MRD was measured by real-time quantitative PCR (RT-qPCR) by determining the levels of the *BCR-ABL*1 fusion transcript, according to international guidelines (7). MRD was defined as the persistence of the *BCR-ABL*1 >0.01% (8). Due to persistently high level of MRD, the patient was started on second-line treatment with Ponatinib for an additional 15 months, failing to achieve MRD negativity. The patient next suffered from a hematological relapse during Ponatinib, with a high rate of blast cells (>60%) in the bone marrow, and was treated with one cycle of chemotherapy according to the hyper-CVAD scheme. Unfortunately, the blast cells were still present in a significant amount (15%). She was then referred to our center and treated with fourth-line therapy with blinatumomab as a bridge to an allogeneic hematopoietic stem cell transplant (HSCT). After the first cycle, the patient achieved a hematological remission withpersistent MRD positivity. After the second cycle, an MRD negativity (<0.01%) was obtained. Therefore, she was referred to receive an allogeneic HSCT from an HLA identical sibling donor. She is incomplete remission (+8 months) and has MRD negativity.

### 2.2 Case n. 2

A 57-year-old male patient was admitted to the Emergency Department due to the onset of evening fever with chills and sweats and isolated thrombocytopenia. Bone marrow aspirate revealed a clonal population of cells (>30%) showing the following immunophenotype with TdT-pos, PAX5-pos, CD10-pos, and CD33-neg. A diagnosis of Ph-negative Bcp-ALL was performed. Therefore, the patient received first-line pediatric-inspired chemotherapy, including Pegylated Asparaginase (PEG-ASP), according to the GIMEMA LAL1913 protocol (9). The patient achieved complete disease remission after the first induction cycle. However, he was switched to blinatumomab due to MRD persistence after the fifth cycle of therapy. MRD was measured by RT-qPCR, and it is defined as the persistence of clonal IgH-rearrangement >10^-4^ (8). Three consecutive cycles were performed, achieving MRD-negativity after the first one and bridging the patient to allogeneic HSCT from a matched unrelated donor (MUD). The patient is currently in complete molecular remission (CMR +6 months after HSCT).

### 2.3 Case n. 3

A 13 years-old female patient was diagnosed with Ph negative Bcp-ALL at the Center of Pediatric Hematology Oncology in our Hospital. She was enrolled in an AIEOP-BFM protocol achieving CR after Induction with persistent MRD positivity. MRD negativity was obtained after consolidation therapy. Five years after achieving CR, a molecular relapse (i.e., a reappearance of the identical IgH-rearrangement >10^-4^) was diagnosed. The patient achieved a second remission after an induction phase containing vincristine, idarubicin, and chrysantaspase due to a previous allergic reaction to PEG-asparaginase. A blinatumomab-cycle was performed because of a persistent MRD positivity, achieving CR with MRD negativity, bridging the patient to an allogeneic HSCT from a sibling HLA-identical donor. She maintained a complete molecular remission with MRD negativity (i.e., clonal IgH-rearrangement <10^-4^ by RT-qPCR) up to the last follow-up in December 2022 (+3 years).

### 2.4 Case n. 4

A 40-year-old female was diagnosed with Ph-positive Bcp-ALL at our Institution. At the diagnosis, we performed a CT scan that showed skeletal lesions of the right shoulder compatible with extramedullary disease (EMD). The patient started on induction therapy with dasatinib and corticosteroids, achieving a major molecular response (*BCR-ABL1* transcript ≤0.1%) (10). Therefore, the patient received an allogeneic HSCT from an HLA-identical sibling donor. The conditioning regimen was myeloablative and based on total-body irradiation (TBI) and cyclophosphamide. Right after, the patient achieved an MRD as early as day +30. Approximately 100 days after the HSCT, the patient experienced an overt relapse, with 40% of blast cells in the bone marrow, a *BCR-ABL1* positivity (39%), and a chimerism with 20% of DNA from the recipient (chimerism was measured by short tandem repeat analysis) (11). Salvage therapy was started with ponatinib and donor lymphocyte infusions (DLIs). At the end of the third DLI, a CR with MRD negativity and a full donor chimerism were achieved. One year later, a new relapse occurred during the maintenance treatment with ponatinib. In the bone marrow, 30% of blast cells was detected, and the mutational analysis by NGS sequencing (12) identified the T315I and the E255V mutation, respectively, in 25% and 60% of *BCR-ABL*1 positive cells. After three cycles of chemotherapy based on the LAL1913 scheme, showing no response, the patient underwent blinatumomab associated with DLI, obtaining a major molecular response after the 1st cycle. After the second cycle, the patient experienced bone pain in the spine and chest, and a CT/PET scan showed lesions compatible with EMD of the ribs and vertebrae. The patient was switched to inotuzumab ozogamcin for 5 cycles without obtaining a response and died of disease progression.

### 2.5 Case n. 5

A 34-year-old female patient with Ph-positive Bcp-ALL was treated as the first line with dasatinib in association with steroids, achieving both complete hematological and molecular remission. After four months of treatment, she experienced an extramedullary relapse of the mammary gland. Therefore, she received rescue therapy with methotrexate and high-dose cytarabine, achieving a new complete response documented by a PET-CT scan. This response was consolidated with allogeneic HSCT from an HLA-identical donor, followed by maintenance therapy with ponatinib. Twelve months after HSCT, the patient suffered a hematological relapse and was treated with vincristine and idarubicin. Due to the persistence of the *BCR-ABL*1 fusion transcript, she underwent therapy with blinatumomab associated with DLIs “escalated dose”. Each DLI was administered after each cycle achieving complete molecular remission after the second DLI course. After the fourth cycle of blinatumomab and DLI, molecular relapse occurred, and the patient was switched to inotuzumab ozogamicin for six cycles reaching CR with MRD negativity. This response was maintained for 6 months, followed by an overt hematologic relapse with 10% leukemic cells in the BM. The patient was enrolled in an experimental trial with CARCIK-CD19 (13), reaching a transient response and, 3 months after the infusion of the cellular product, had an overt hematological relapse and died of disease progression.

### 2.6 Case n. 6

A 14-year-old male presented to the Center of Pediatric Hematology Oncology in our Hospital, reporting spinal pain and pancytopenia. He was diagnosed with a Ph-negative Bcp-ALL and enrolled in the ongoing protocol AIEOP-BFM ALL 2017. During the induction phase, he experienced a severe adverse event characterized by septicemia due to carbapenemase-producing Klebsiella pneumoniae (KPC-KP) and fungemia due to Candida species. These infections were complicated by pneumonia requiring positive pressure ventilation and a cerebral abscess. The patient received treatment with ceftazidime/avibactam, voriconazole, and supportive therapy until the resolution of the infectious complications. Because of the persistence of MRD after the induction treatment, the patient received therapy with blinatumomab for 4 cycles in an off-label manner because of the patient’s age (<18 years), reaching MRD negativity after the first one. The patient was then followed with periodical BM assessments, maintaining complete remission up to two years after the completion of the treatment.

### 2.7 Case n. 7

A 16-year-old male patient was diagnosed with a Ph-negative Bcp-ALL at the Center of Pediatric Hematology Oncology in our Hospital. He was enrolled in the ongoing protocol (AIEOP-BFM ALL 2017), achieving complete remission with MRD negativity at the end of consolidation (final risk: medium). Two months after the end of the first-line treatment, an early (<30 months from diagnosis) bone marrow isolated relapse was diagnosed. Thus, the patient received a second line therapy based on international protocol IntReALL-2010 HR (including mitoxantrone, ASP, and vincristine), achieving CR with the persistence of MRD positivity. For this reason, because he reached adult age, he received treatment with blinatumomab, completing 2 cycles and achieving MRD clearance after the first cycle. Then, he underwent an allogeneic MUD-HSCT in another Center. One year after the completion of the therapy, the patient is still in CR withMRD negativity.

## 3 Results: T-cell lymphocyte kinetics at baseline and after one blinatumomab cycle

**Figure 1:**
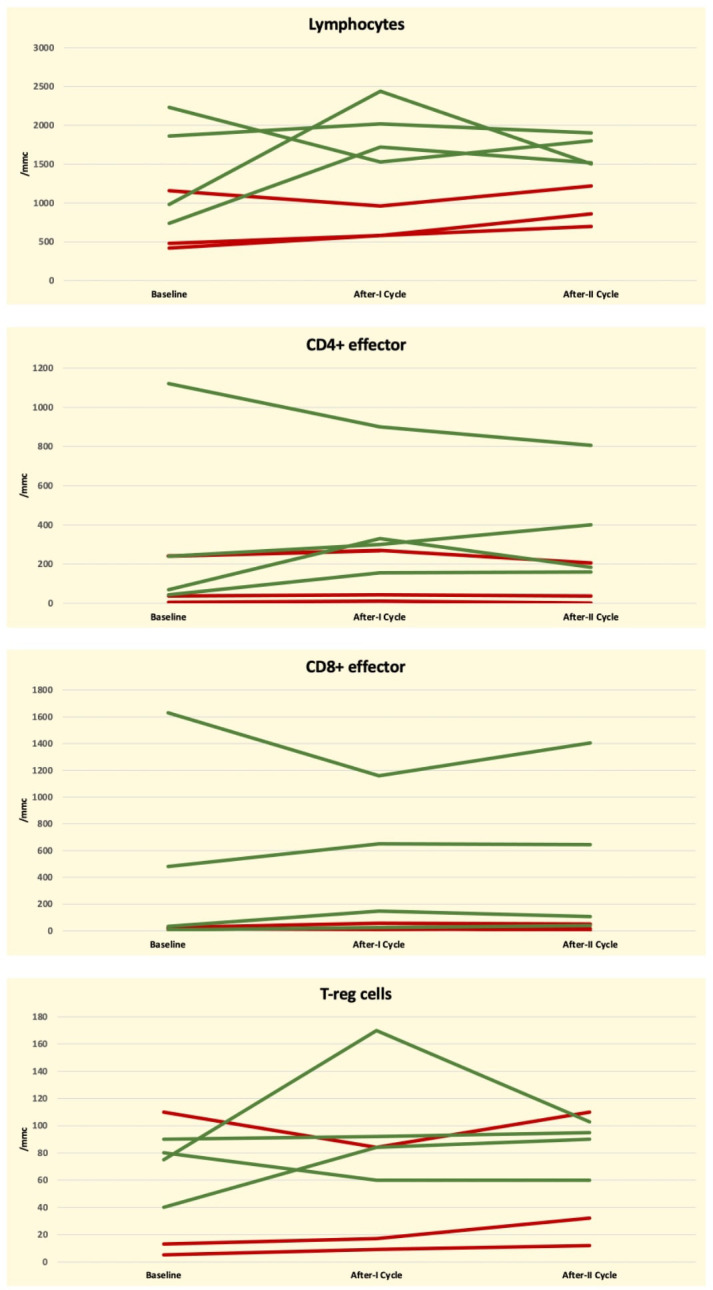
Graphical representation of Global T-Cell lymphocyte kinetics at baseline and following the first blinatumomab cycle according to MRD negative (green line) and positive (red line) status in seven ALL-B patients.

Patients’ peripheral blood samples (PB) were evaluated using flow cytometry at baseline and after the first blinatumomab cycle, according to our center’s internal guidelines. Global T-lymphocyte kinetics was assessed by measuring the absolute counts of CD3+, CD4+ effector memory (T4-EM), CD8+ effector memory (T8-EM) T-lymphocytes, and the count of T-regulatory cells (T-reg). The individual lymphocyte kinetic variations were reported for each patient. The T-cell kinetics between different populations is represented in **Figure 1**. Patients were divided into two groups according to the MRD status following the first blinatumomab cycle (positive in three vs. negative in four patients). MRD status was chosen as the primary endpoint because it has been highlighted as the most powerful prognostic factor for patients with Bcp-ALL (14). The working hypothesis of the present study was that the status of immune-mediators, such as the T-cell subsets, their ability to express cytokines involved in the anti-tumoral response, and the expression of the T-cell exhaustion markers could potentially be correlated to the depth of blinatumomab response.

In order to evaluate any possible correlation and given the heterogeneity of the patients in terms of disease type (Ph-Positive versus Ph-Negative), prior treatment (chemotherapy versus chemo-free, HSCT versus no HSCT), and tumor burden (MRD positive versus hematological relapse), we measured both the absolute counts of the immune-cell subsets and the ratio between the counts after the first cycle of blinatumomab (T0) and at baseline (T1). The absolute values and the ratio T1/T0 of the different classes of lymphocytes are reported in **Table 2**.

**Table 2:** Reported count of peripheral blood lymphocytes evaluated at baseline and after the first cycle of blinatumomab, expressed through absolute count and ratio (T1/T0).

Case N.	Lymphocytes			CD4			CD4-IFNγ			CD4-PD1			CD8			CD8-IFNγ			CD8-PD1			T-reg			MRD Response after I cycle
	Baseline	I cycle	*Ratio*	Baseline	I cycle	*Ratio*	Baseline	I cycle	*Ratio*	Baseline	I cycle	*Ratio*	Baseline	I cycle	*Ratio*	Baseline	I cycle	*Ratio*	Baseline	I cycle	*Ratio*	Baseline	I cycle	*Ratio*	
1	1160	960	*0.83*	240	270	*1.13*	215	235	*1.09*	4.79	2.74	*0.57*	16	10	*0.63*	267	273	*1.02*	2.25	1.23	*0.55*	110	84	*0.76*	Positive
2	2230	1530	*0.69*	1120	900	*0.80*	320	300	*0.93*	2.4	1.9	*0.79*	1630	1160	*0.71*	1081	879	*0.81*	1.9	1.6	*0.84*	80	60	*0.75*	Negative
3	1860	2020	*1.09*	240	300	*1.25*	51	191	*3.74*	1.54	1.52	*0.99*	480	650	*1.35*	95	212	*2.23*	2	1.1	*0.55*	90	92	*1.02*	Negative
4	480	580	*1.21*	5	11	*2.2*	NA	NA	*NA*	NA	NA	*NA*	10	20	*2*	NA	NA	*NA*	NA	NA	*NA*	5	9	*1.80*	Positive
5	420	580	*1.38*	38	44	*1.16*	NA	NA	*NA*	NA	NA	*NA*	25	57	*2.28*	NA	NA	*NA*	NA	NA	*NA*	13	17	*1.31*	Positive
6	980	2440	*2.49*	70	330	*4.71*	35	166	*4.74*	1.7	1.51	*0.89*	33	146	*4.24*	76	244	*3.21*	2.8	2.64	*0.94*	75	170	*2.27*	Negative
7	740	1720	*2.32*	44	155	*3.52*	48	294	*6.13*	1.52	1.23	*0.81*	6	24	*4*	72	381	*5.29*	2.2	1.3	*0.59*	40	84	*2.10*	Negative

NA stands for "not available".

We compared the median absolute cell counts at baseline and after the first cycle and the ratio T1/T0 between MRD-positive and MRD-negative patients (respectively, MRD-responsive and MRD not-responsive patients) at the end of the first cycle of blinatumomab. These unpaired comparisons were performed by the Mann-Whitney U-test, with a p <0.05 considered significant.

The MRD-responsive patients after the 1^st^ cycle had a median baseline count of T- lymphocytes of 1450/mmc, that was higher than that of the MRD not-responsive patients. The statistical comparison of the absolute cell counts between MRD-negative and MRD-positive patients did not reach statistical significance (p = 0.06). Similarly, the median values of T4-EM (368 vs 94), T8-EM (537 vs. 17), and T-reg (71 vs. 39) were higher in MRD responsive patients than in MRD-not responsive patients but without reaching statistical significance (0.22, 0.14, and 0.40, respectively). The p-values obtained by comparing the ratio T1/T0 measured in MRD-responsive and in MRD not-responsive patients were, respectively, 0.86 for absolute T-lymphocyte, T8-EM, and T-reg and 0.63 for T4-EM.

To evaluate cytokines involved in the anti-tumoral response, we measured the expression of interferon γ (IFNγ) and programmed death protein-1 (PD1) in T4-EM and T8-EM. These data were collected only from 5 patients, 4 of them were MRD-responsive; thus, an accurate analysis could not be performed. However, the preliminary results are reported in **Table 2**.

Based on these findings, we assume that the higher value of total lymphocytes can predict a deep and robust response to blinatumomab, despite not achieving a statistically significant correlation. As for the specific subsets of the T-cell population and their expression of IFNγ and PD1, a minimal number of heterogeneous patients makes it difficult to draw conclusions.

## 4 Discussion

Ever since the development of the first-in-class bispecific T-cell engager antibody blinatumomab, the importance of T-cell kinetics was discussed as a hypothetically predictive factor for MRD response, although limited data are available. In a phase 2 study of blinatumomab, Zugmaier and colleagues assessed the long-term survival of 36 adult Bcp-ALL relapsed/refractory (RR) patients (15). Twenty-five patients (69%) achieved MRD response, and ten were long-term survivors with overall survival (OS) greater than 30 months, including both patients that were consolidated with allogeneic HSCT and blinatumomab treatment alone for a total of 5 cycles. The more significant expansion of CD3+ T-cells and increased numbers of CD3+ effector memory cells were predominant in the long-term survivors in both cycle 1 and cycle 2. The OS was inferior for 30 months in patients with persistent MRD positivity.

Nägele et al, investigated the correlation between immunological biomarkers and the clinical response to blinatumumab in the same study population. In this study the authors, by monitoring serum cytokines before and during the first week of each course of blinatumomab, demonstrated that in patients in complete remission after blinatumomab the serum levels ​​of IL-6, IL-10, and IFNγ reach higher values than in non-responders patients (16).

In a phase 1 dose-escalated study, the same authors demonstrated a correlation between a greater expansion of CD4+ and CD8+ T-cells and the clinical response to blinatumomab (17).

Finally, in the phase-3 trial leading to the approval of blinatumomab in relapsed/refractory Bcp-ALL, the percentage of CD3+ T-cells measured at baseline had a significant impact on MRD-response to blinatumumab and greater values of both CD4+ or CD8+ T-cells at baseline predicted higher rates of hematological remission (18).

As regard the potential role of the T-cell exhaustion in hampering the response of Bcp-ALL to blinatumomab, Feucht and co-workers have demonstrated that blast cells of Bcp-ALL highly expressing PD1-ligand are less susceptible to the blinatumomab-induced cell lysis and this phenomenon could be reversed, *in vitro*, by the adjunction of PD1-inhibitors (19).

The role of MDSCs, on the other side, is increasingly discussed and known, also in the setting of Bcp-ALL and the relationship with the T-lymphocyte compartment (20). Zahran et al. reported that the MDSCs correlated to the missed therapeutic response and wished for a future role as a prognostic indicator or a potential therapeutic target (21). The same results were confirmed in pediatric patients, with granulocytic MDSCs levels correlated positively with therapeutic responses and Bcp-ALL disease prognostic markers (among which MRD) (6).

The major limitations of the present study are the very limited number and the heterogeneity of the patients, which makes it impossible to draw conclusions for the clinical practice.

Moreover, although the recognized role of the MDSCs in mediating the immune evasion of Bcp-ALL, the investigation of the MDSCs in hampering the activity of blinatumomab was not included in the initial conceptualization of the present study and was included in a subsequent revision of the study-design that is still ongoing. This knowledge, together with that of the lymphocyte kinetics, will allow us to have a better understanding of the underlying mechanisms of the sensitivity of the Bcp-ALL to immune therapy.

Mainly because of these limitations, the statistical analysis failed to demonstrate an association between the T-cell kinetics and the MRD response after one cycle of blinatumomab. However, the correlation between the absolute lymphocyte counts at baseline and the MRD response to Blinatumomab was close to the statistical significance threshold in such a small sample of patients. This is in line with what has already been reported (18) and is encouraging in continuing the study by expanding the cohort of the patients in order to assess not only the function of the T-cell subsets but also the potential impact of MDSCs in influencing the lymphocytes-compartment and the response to blinatumomab.Given that the T-cell expansion in response to blinatumomab could be a biological pre-requisite for the anti-leukemic activity, we also evaluated the T1/T0 ratio rather than the absolute lymphocyte counts alone. In patients with relapsed/refractory Bcp-ALL, a low pre-treatment lymphocytes count, a non-permissive microenvironment due to MDSCs, or a low T1/T0 ratio during the 1^st^ cycle, could direct the choice toward different drugs (i.e., antiCD22 inotuzumab ozogamicin) (22).

Finally, a strict correlation between MDSCs and PD1/PD1 ligand was reported (23). In different models, MDSCs could contribute to the resistance to immune checkpoint inhibition drugs by inhibiting the anti-neoplastic properties of T and NK cells and stimulating T-regs (24).

Furthermore, IFN-γ could regulate the role and the function of the MDSCs through the modulation of the anti-apoptotic Bcl2 protein by direct interaction with the phosphorylated STAT-1 (25). Based on these findings, monitoring peripheral MDSCs (defined as CD11b+CD14−CD15+ or CD11b+CD14−CD66b+) (26) and investigation of their potential inhibition in improving the T-lymphocyte role, represents our future aim for the prosecution of the study.

## 5 Conclusion

In the era of the anticancer immunotherapy the discovery of immunological biomarkers linked to the clinical response to the bi-specific antibodies could potentially lead to a better selection of the patients likely to benefit from the treatment. A large, prospective trial could provide this data, driving the physician toward a choice that should be adapted to the health of the patient’s immune system.

## Data availability statement

The original contributions presented in the study are included in the article/supplementary material. Further inquiries can be directed to the corresponding authors.

## Ethics statement

The studies involving humans were approved by Comitato Etico Policlinico “G.Rodolico-San Marco” Catania 1. The studies were conducted in accordance with the local legislation and institutional requirements. The participants provided their written informed consent to participate in this study. Written informed consent was obtained from the individual(s) for the publication of any potentially identifiable images or data included in this article.

## Author contributions

AD, UM, SL, and AR contributed to the conception and design of the study. AD, NP, SL, and UM organized the database. AD performed the statistical analysis. AD, UM, and SL wrote the first draft of the manuscript. EM, MP, CV, GM, PF, LN, and CM collected literature and patient data. AD, UM, AR, SL, and FDR revised the manuscript. All authors contributed to the article and approved the submitted version.
